# Assessing sustained uptake of latrine and child feces management interventions: Extended follow-up of a cluster-randomized controlled trial in rural Bangladesh 1–3.5 years after intervention initiation

**DOI:** 10.1016/j.ijheh.2023.114149

**Published:** 2023-05

**Authors:** Mahfuza Islam, Mahbubur Rahman, Mohammad Abdullah Heel Kafi, Leanne Unicomb, Mostafizur Rahman, Andrew Mertens, Jade Benjamin-Chung, Benjamin F. Arnold, John M. Colford, Stephen P. Luby, Ayse Ercumen

**Affiliations:** a Environmental Intervention Unit, Infectious Disease Division, icddr,b, Dhaka, Bangladesh; b School of Population and Public Health, University of British Columbia, Vancouver, Canada; c Division of Epidemiology and Biostatistics, University of California, Berkeley, CA, USA; d Francis I. Proctor Foundation, University of California, San Francisco, CA, USA; e Woods Institute for the Environment, Stanford University, Stanford, USA; f Department of Forestry and Environmental Resources, North Carolina State University, Raleigh, NC, USA

**Keywords:** Sanitation, Open defecation, Latrine, Potty, Sani-scoop, Child feces management, User uptake

## Abstract

**Background:**

Sanitation interventions typically result in modest increases in latrine access, and any gains in latrine access and use are often not sustained over time. Sanitation programs also rarely include child-focused interventions such as potties. We aimed to assess the sustained effect of a multi-component sanitation intervention on access to and use of latrines and child feces management tools in rural Bangladesh.

**Methods:**

We conducted a longitudinal substudy nested within the WASH Benefits randomized controlled trial. The trial provided latrine upgrades, child potties and sani-scoops for feces removal, along with behavior change promotion to encourage use of the delivered hardware. Promotion visits to intervention recipients were frequent during the first 2 years after intervention initiation, decreased in frequency between years 2–3, and ceased after 3 years. We enrolled a random subset of 720 households from the sanitation and control arms of the trial in a substudy and visited them quarterly between 1 and 3.5 years after intervention initiation. At each visit, field staff recorded sanitation-related behaviors through spot-check observations and structured questionnaires. We assessed intervention effects on observed indicators of hygienic latrine access, potty use and sani-scoop use and investigated whether these effects were modified by duration of follow-up, ongoing behavior change promotion and household characteristics.

**Results:**

The intervention increased hygienic latrine access from 37% among controls to 94% in the sanitation arm (p < 0.001). Access among intervention recipients remained high 3.5 years after intervention initiation, including periods with no active promotion. Gains in access were higher among households with less education, less wealth and larger number of residents. The intervention increased availability of child potties from 29% among controls to 98% in the sanitation arm (p < 0.001). However, fewer than 25% of intervention households reported exclusive child defecation in a potty or had observed indicators of potty and sani-scoop use, and gains in potty use declined over the follow-up period, even with ongoing promotion.

**Conclusion:**

Our findings from an intervention that provided free products and intensive initial behavior change promotion suggest a sustained increase in hygienic latrine access up to 3.5 years after intervention initiation but infrequent use of child feces management tools. Studies should investigate strategies to ensure sustained adoption of safe child feces management practices.

## 1 Introduction

Safely managed sanitation services are available to 62% of urban populations but only 44% of rural populations worldwide (WHO/UNICEF, 2021). If the current trends persist, 2.8 billion people will lack safely managed sanitation services by 2030 (WHO/UNICEF, 2021). Access to sanitation is considered a primary barrier against fecal-orally transmitted diseases. However, the effectiveness of sanitation improvements on interrupting disease transmission depends on their coverage and sustained adoption by users. Several sanitation trials have found either no impact or mixed impact of sanitation interventions on child diarrhea and growth (Arnold et al., 2010; Briceño et al., 2015; Clasen et al., 2014; Fenn et al., 2012; Patil et al., 2014; Pickering et al., 2015; Pickering et al., 2019). One possible reason could be that the sanitation interventions in these studies may not have increased latrine use sufficiently to reduce exposure to fecal pathogens (Clasen et al., 2014).

A systematic review and meta-analysis of sanitation intervention studies estimated that the interventions increased latrine coverage on average by 14% and latrine use by 13% (Garn et al., 2017). Even among households with latrine access, open defecation is often still practiced by household members (Barnard et al., 2013; Coffey et al., 2014). While approaches focused on behavior change such as the Community-Led Total Sanitation (CLTS) campaign can significantly increase latrine access and reduce open defecation (Harvey, 2011; Kar and Chambers, 2008; Mosler, 2012), problems persist with the long-term sustainability of latrines such as maintenance and repairs after initial installation (Mosler et al., 2018). Fecal sludge management remains an additional problem in the absence of mechanisms to hygienically transport fecal waste away from communities and treat it before being discharged into the environment. In low-income countries, >70% of fecal waste flows into the environment untreated (World Bank, 2011). Additionally, young children often continue to defecate directly in the living environment even when latrines are present (Islam et al., 2018). For example, in rural Bangladesh, more than 75% of young children defecate in the open despite widespread access to on-site latrines, and child feces are unhygienically disposed of within the domestic environment by the majority of households (Ercumen et al., 2018; Islam et al., 2018, 2020). Child-focused interventions such as potties are rarely included in conventional sanitation programs.

In this study, we present data from a substudy nested within the sanitation and control arms of a randomized controlled trial in rural Bangladesh (WASH Benefits) that provided latrine upgrades and child feces management tools to intervention households. We used longitudinal data collected between 1 and 3.5 years after the initiation of the interventions to assess their sustained impact on indicators of latrine access, use and maintenance, and use of child feces management tools. We also investigated effect modification of intervention impacts on these indicators by time since study onset, ongoing behavior change promotion and household factors.

## 2 Methods

### 2.1 Study design

WASH Benefits was a cluster randomized controlled trial of water, sanitation, hygiene and nutrition interventions with 5551 participating compounds in rural villages in the Gazipur, Kishoreganj, Mymensingh, and Tangail districts of central Bangladesh (ClinicalTrials.gov NCT01590095) (Arnold et al., 2013; Luby et al., 2018). The study areas were chosen to not have any ongoing government or NGO programs on water, sanitation, hygiene (WASH) or nutrition. The trial enrolled compounds with a pregnant woman in her first or second trimester. Six to eight adjacent enrolled compounds were grouped into a cluster, and eight adjacent clusters were grouped into a study block. Clusters within each block were randomized to one of six intervention arms or into a double-sized control arm resulting in geographically pair-matched clusters of intervention and control compounds. A 1-km buffer was enforced between clusters to minimize spillovers; analysis of trial data found no evidence of spillover between clusters in different arms of the trial (Luby et al., 2018). The trial followed the birth cohort born to the enrolled pregnant women (referred to as “index children” hereafter) for two years to measure diarrhea and child growth. Further details of the study design have been previously described (Arnold et al., 2013).

### 2.2 Sanitation interventions of the WASH Benefits trial

The sanitation intervention was implemented at the compound level (group of households shared by extended families). All households in the enrolled compound received upgrades to double-pit pour-flush latrines, with labor and minor financial contributions provided by households. If the index household (household where the index child lived) did not have a latrine, a latrine was constructed; for non-index households in the compound, existing latrines were upgraded. The latrines included two pits with five concrete rings per pit, a slab, water seal and a superstructure for privacy. The dual-pit design allows alternating use of pits so that the contents of the inactive pit can undergo pathogen inactivation and be safely emptied by household members and/or used as soil amendment. The slab and the water seal provide a barrier between latrine users and fecal material and also minimize flies. In the enrolled compounds, each household with children <5 years also received one child potty, and each household received one sani-scoop for removal and safe disposal of child and animal feces. Further details of the interventions have been described elsewhere (Arnold et al., 2013).

Hardware was delivered within a behavior change program, which consisted of periodic household visits from local community health promoters trained by the trial staff. The promotion visits primarily targeted the index household but other households in enrolled compounds were encouraged to attend. The visits included sharing messages using pictorial guides and conversations with the index child's caregivers. These activities were designed to encourage participants to correctly and consistently use the provided sanitation hardware and adopt safe sanitation practices in their daily lives (Dreibelbis et al., 2013). Messaging about latrine use focused on exclusive latrine use by all adults and older children in the compound and latrine training for young children. Households were informed to transfer the latrine superstructure (walls and roof) to the second pit when the first pit became full, but no messaging was given on pit emptying and maintenance. Messaging about child feces management included potty use for children aged 6 months and older until they stared to use the latrine, safe disposal of child feces into the latrine from the potty or from the courtyard using the sani-scoop immediately after defecation, and removal of animal feces from the courtyard using the sani-scoop.

Promoters visited intervention households intensively during the first two years after intervention initiation, with six visits per month on average. Promotion frequency was reduced to one visit per month at the end of the second year, gradually tapered down during the third year and ceased at the end of the third year. Promoters did not visit households in the control arm. Further details of intervention implementation have been described elsewhere (Unicomb et al., 2018). The parent trial measured intervention uptake 1–2 years after intervention initiation. In the sanitation arm, >95% of households had a latrine with a functional water seal, children in 54% of households were observed to defecate in a potty or hygienic latrine, 27% of households used the sani-scoop to handle human feces and 15% to handle animal feces (Luby et al., 2018, Parvez et al., 2018). These measurements only focused on a few key sanitation indicators as they covered all intervention arms and did not extend beyond 2 years after intervention initiation.

### 2.3 Longitudinal substudy

We conducted a longitudinal substudy among a randomly selected subset of households enrolled in the sanitation and control arms of the WASH Benefits study to leverage the design and infrastructure of the large-scale randomized controlled trial. Four households per cluster were randomly selected from each sanitation cluster and from one of two control clusters in the same block, maintaining the pair-matched design of the parent trial and resulting in a sample size of 720 households (360 per arm). Households were eligible for enrollment in the substudy if the index child was alive and available or if there was another child <24 months available in the household in the case of index child death.

We visited households enrolled in the substudy approximately quarterly for a total of 8 visits between 1 and 3.5 years after intervention initiation by the parent trial. Therefore, the substudy period captured approximately one year (substudy visits 1–3) with intensive promotion activities among intervention recipients, one year (substudy visits 4–6) with less intensive promotion and six months (substudy visits 7–8) with no promotion. At each visit, field staff observed indicators of access to and use of latrines, potties and sani-scoops through spot check observations and also recorded self-reported latrine use, maintenance, sharing and pit emptying practices, and self-reported use of potties and sani-scoops/similar tools through structured questionnaires. Latrine use was separately reported for adults and child age groups with different expected defecation practices (<3 years, 3–8 years, 8–15 years).

### 2.4 Composite sanitation indicators

We combined the spot-check observations for each individual data collection round to generate composite binary indicators for (1) hygienic latrine access, (2) potty use and (3) sani-scoop/similar tool use. A household was defined as having access to a hygienic latrine if the primary latrine was observed to have a functional water seal, feces were contained within a septic tank/pit and there were no visible feces on the slab or floor of the latrine. We defined a household as having indicators of potty use if a potty was observed to be present, accessible by caregivers and appeared recently used. We classified the potty as accessible if the caregiver could deliver it within 30 s without assistance to field staff standing where the child usually defecates; we chose 30 s as a reasonable estimate for how quickly a regularly used item can be retrieved. We classified the potty as recently used if it was observed to be either currently wet, or dry and clean (i.e., not covered with dust). We defined a household as having indicators of sani-scoop/similar tool use if a sani-scoop or other feces removal tool was observed to be present, accessible by adults and appeared recently used. We classified the tool as accessible if it was located in or near the courtyard and could be retrieved without assistance, and as recently used based on the same visual indicators as for the potties. We did not include reported behaviors in the composite indicators as they may be subject to biased reporting but analyzed them separately as individual outcomes.

### 2.5 Data analysis

To assess the impact of the sanitation intervention on latrine, child potty and sani-scoop/similar tool access and use, we compared the prevalence of each individual and composite spot-check indicator and reported practice between the sanitation and control arms, using pooled data from all eight data collection rounds. We used population-averaged generalized linear models (GLM) (Hubbard et al., 2010) with a Gaussian error distribution and identity link to estimate prevalence differences (PDs) (McNutt et al., 2003), using robust standard errors at the block level. The study block represents the highest level of outcome clustering in our trial, and robust standard errors by block account for outcome correlation across multiple levels in the study, including households within study blocks and multiple visits within households. We adjusted estimates for study block to account for the geographical pair-matching. We did not include any additional adjustment covariates in our models as the randomized study design led to well-balanced study groups. We conducted a Cochran-Armitage test for each composite indicator versus data collection round (separately within each study arm) to assess any linear time trends and also qualitatively compared the prevalence of each composite indicator at each individual data collection round between study arms. As a measure of consistency of access and use, we tabulated during how many of the eight data collection rounds a given household met the composite indicators for hygienic latrine access, potty use, and sani-scoop use.

We investigated effect modification on intervention impacts on hygienic latrine access, potty use and sani-scoop/similar tool use by the following factors: time since study onset, ongoing intervention promotion, age of index child and index child's primary caregiver, education of caregiver, education of father, household wealth, number of individuals in the compound, and number of children <5 years in the compound. For the time since study onset, we used a binary indicator variable for the first half (data collection rounds 1–4) vs. second half (rounds 5–8) of the follow-up period. We also compared periods of ongoing intervention promotion (rounds 1–6) vs. no promotion (rounds 7–8). We calculated a household wealth index using principal components analysis based on measured assets and housing materials (Howe et al., 2012; Vyas and Kumaranayake, 2006), including the following: presence of electricity; number of wardrobe, table/chair/bench, bed, television, refrigerator, motorcycle, sewing machine, mobile phones; materials of the wall, roof and floor of the house; and main fuel used for cooking. Based on this index, we defined a binary indicator variable for above-versus below-median wealth. For the continuous and categorical effect modification variables, we examined the empirical data distribution and determined a cut-off that corresponds to the median to generate binary indicators for the effect modification analysis. We included additive interaction terms between study arm and these binary variables in our linear models, and we interpreted p-values <0.2 for the interaction term as evidence for effect modification.

### 2.6 Ethical considerations

All households provided written informed consent. The protocol was reviewed and approved by human subjects review committees at the International Centre for Diarrhoeal Disease and Research, Bangladesh (icddr,b) (PR-11063), University of California, Berkeley (2011-09-3652), and Stanford University (25863).

## 3 Results

We enrolled 720 households starting in June 2014 and completed data collection in December 2016. Randomization balanced baseline characteristics between households in the sanitation and control arms (Table 1). Most households (80%) participated in all eight data collection rounds, while 10% of households completed seven rounds, 4% completed six rounds, and 6% completed between one and five rounds. The percent of households completing all rounds was slightly higher in the sanitation arm (83%) than in the control arm (76%). Households lost to follow-up vs. remaining in the study had similar baseline characteristics (Contreras et al., 2021).

**Table 1:** Baseline characteristics among enrolled households in rural Bangladesh.

Household characteristics	Sanitation N = 360 %(n)	Control N = 360 %(n)
Respondent's age in years, mean (SD)	24 (5)	24 (5)
Mother's education		
No or primary	56 (201)	56 (200)
Secondary or above	44 (159)	44 (160)
Father's education		
No or primary	41 (148)	43 (154)
Secondary or above	59 (211)	57 (206)
Number of rooms in household, mean (SD)	1.9 (1.1)	2.0 (1.28)
Number of households in compound^a^, mean (SD)	2.6 (1.65)	2.4 (1.38)
Number of children <3 years in household, mean (SD)	1.3 (0.46)	1.3 (0.54)
Number of children <3 years in compound, mean (SD)	2.0 (1.26)	2.0 (1.12)
Households with:		
Natural wall (made by jute/bamboo/mud)	28 (100)	34 (124)
Electricity	60 (216)	57 (205)
Cell phone	87 (313)	86 (309)
Television	33 (121)	31 (113)

SD: Standard deviation.

^a^**Compound** is a group of households around a central courtyard shared by extended families.

### 3.1 Observed indicators of access and use

All indicators of latrine, child potty and sani-scoop/similar tool access and use were significantly higher in households in the sanitation intervention arm versus controls pooling observations from all eight data collection rounds over the 2.5-year follow-up period (Table 2). In the sanitation arm, 94% of households had access to a hygienic latrine compared to 37% of controls (prevalence difference [PD]: 57.6%, 95% CI: 52.0–63.3, p < 0.001) (Table 2). Hygienic latrine access was also more consistent in the sanitation arm compared to controls; 65% of sanitation arm households had access to a hygienic latrine during all eight data collection visits compared to 13% of control households, and 2% of sanitation arm households did not have hygienic latrine access during any visit compared to 44% of control households (Fig. S1).

**Table 2:** Observed indicators of access and use for hygienic latrines and child feces management tools by study arm 1–3.5 years after intervention initiation.

	Sanitation N = 2735^a^ % (n)	Control N = 2658^a^ % (n)	PD^b^ (95% CI)	p-value
**Hygienic latrine access**				
Functional water seal	96.6 (2644)	39.4 (1048)	57.4 (51.8, 63.0)	<0.001
Feces well-contained within a septic tank or pit	98.1 (2673)	21.0 (559)	77.2 (72.9, 81.6)	<0.001
No visible feces on the slab or floor	98.0 (2679)	89.4 (2341)	8.7 (5.6, 11.8)	<0.001
Composite indicator (all three above)	94.4 (2583)	36.9 (981)	57.6 (52.0, 63.3)	<0.001
**Child potty use**				
Potty present	97.7 (2672)	29.1 (771)	68.8 (63.3, 74.5)	<0.001
Accessible by mother/caregiver^c^	20.7 (566)	4.3 (115)	16.4 (12.6, 20.3)	<0.001
Appeared recently used^d^	58.6 (1603)	11.6 (316)	47.0 (42.2, 51.9)	<0.001
Composite indicator (all three above)	15.3 (418)	3.5 (93)	11.9 (8.8, 15.0)	<0.001
**Sani-scoop use**				
Sani-scoop or other feces removal tool present	99.6 (2723)	88.7 (2354)	10.9 (8.2, 13.5)	<0.001
Accessible by adult^e^	39.3 (1060)	23.2 (543)	16.1 (5.1, 27.1)	0.004
Appeared recently used^d^	56.3 (1520)	51.5 (1202)	4.9 (0.1, 9.5)	0.04
Composite indicator (all three above)	22.1 (605)	12.9 (342)	9.5 (2.2, 16.7)	0.01

PD: Prevalence difference; CI: Confidence interval.

a Pooled data from eight data collection rounds over 2.5 years.

b Estimated using robust standard errors for repeated measures and geographical clustering.

c Can be retrieved within 30 s without assistance when standing where child usually defecates.

d Currently wet, or dry and clean, i.e., not covered with dust.

e Located in or near the courtyard and can be retrieved without assistance.

The intervention increased child potty presence from 29% among controls to 98% in the sanitation arm (PD: 68.8, 95% CI: 63.3–74.5, p < 0.001), while most households in both arms had a tool that can be used for feces removal (Table 2). Intervention households had higher levels of visual indicators for both child potty and sani-scoop/similar tool use compared with controls, but their use was ultimately low even in intervention households. In the sanitation arm, 15% of households had indicators of child potty use compared to 4% of controls (PD: 11.9, 95% CI: 8.8–15.0, p < 0.001). No households in either arm had indicators of child potty use during all visits, while 39% of sanitation households and 89% of control households did not have indicators of child potty use during any visit (Fig. S2). In the sanitation arm, 22% of households had indicators of sani-scoop/similar tool use while 13% of controls had indicators of a feces removal tool use (PD: 9.5, 95% CI: 2.2–16.7, p = 0.01) (Table 2). Only 2% of sanitation arm households and none of the control households had indicators of sani-scoop/similar tool use during all eight visits, and 46% of sanitation arm households did not have indicators of sani-scoop/similar tool use during any visit compared to 61% of control households (Fig. S3).

### 3.2 Reported use and maintenance

Reported exclusive latrine use for defecation by adults increased from 77% among controls to 86% in the sanitation arm (PD: 8.8, 95% CI: 4.1–13.6, p < 0.001) and latrine use by children 3–8 years old increased from 43% to 61% (PD: 17.4, 95% CI: 10.9–23.9, p < 0.001); the intervention did not significantly impact latrine use by children <3 years or 8–15 years old (Table 3). In the sanitation arm, 19% of households shared their primary latrine with other households compared to 52% of controls (PD: −33.5, 95% CI: −40.0–27.0, p < 0.001) (Table 3). Only 4% of sanitation arm households reported emptying the pit of their primary latrine in the time between two successive data collection visits vs. 23% of controls. In both arms, approximately two thirds of households reported burying pit contents, 30% reported discharging the waste into a water body and the rest reported releasing it into fields.

**Table 3:** Reported use of latrines and child feces management tools by study arm 1–3.5 years after intervention initiation.

	Sanitation N = 2735^a^ % (n)	Control N = 2658^a^ % (n)	PD^b^ (95% CI)	p-value
Exclusive latrine use for defecation				
Adults	86.2 (2357)	77.4 (2056)	8.8 (4.1, 13.6)	<0.001
Children <3 years	3.5 (80)	2.7 (58)	0.8 (−0.7, 2.3)	0.27
Children 3–8 years	60.6 (949)	43.3 (644)	17.4 (10.9, 23.9)	<0.001
Children 8–15 years	88.8 (1225)	84.8 (1055)	4.0 (−1.1, 9.1)	0.12
Latrine sharing				
Latrine shared with other households	18.7 (511)	52.1 (1384)	−33.5 (−40.0, −27.0)	<0.001
Latrine maintenance and pit emptying				
Latrine not maintained/cleaned	0.1 (2)	6.2 (164)	−6.2 (−8.2, −4.2)	<0.001
Emptied pit since previous visit	3.5 (89)	23.2 (616)	−19.3 (−23.0, −15.7)	<0.001
Potty use (for children <3 years)				
Last child defecation in potty	41.1 (1125)	9.3 (247)	31.9 (27.6, 36.3)	<0.001
Child always uses potty for defecation	18.9 (518)	4.8 (128)	14.2 (10.3, 17.8)	<0.001
Child feces disposed of in latrine	58.7 (1607)	11.3 (301)	47.4 (42.3, 52.6)	<0.001
Sani-scoop/feces removal tool use				
Scoop/tool used every day for feces disposal	89.2 (2440)	73.7 (1961)	15.6 (10.1, 20.6)	<0.001
Scoop/tool used to handle child feces	52.2 (1435)	71.1 (1899)	−18.9 (−24.5, −13.3)	<0.001

PD: Prevalence difference; CI: Confidence interval.

a Pooled data from eight data collection rounds over 2.5 years.

b Estimated using robust standard errors for repeated measures and geographical clustering.

In the sanitation arm, 41% of households reported last defecation by children <3 years in a potty compared to 9% of controls (PD: 31.9, 95% CI: 27.6–36.3, p < 0.001) and 19% reported exclusive potty use for defecation by children <3 years compared to 5% of controls (PD: 14.2, 10.3–17.8, p < 0.001) (Table 3). Among households in the sanitation arm, 59% reported disposing of child feces in the latrine compared to 11% of controls (PD: 47.4, 95% CI: 42.3–52.6, p < 0.001). Households in the sanitation arm were also significantly more likely to report using a sani-scoop or similar feces removal tool every day for some form of feces disposal (PD: 15.6, 95% CI: 10.1–20.6, p < 0.001); however, reported use to specifically handle child feces was lower in the sanitation arm compared to controls (PD: −18.9, 95% CI: −24.5, −13.3, p < 0.001) (Table 3).

### 3.3 Time trends

**Fig. 1:**
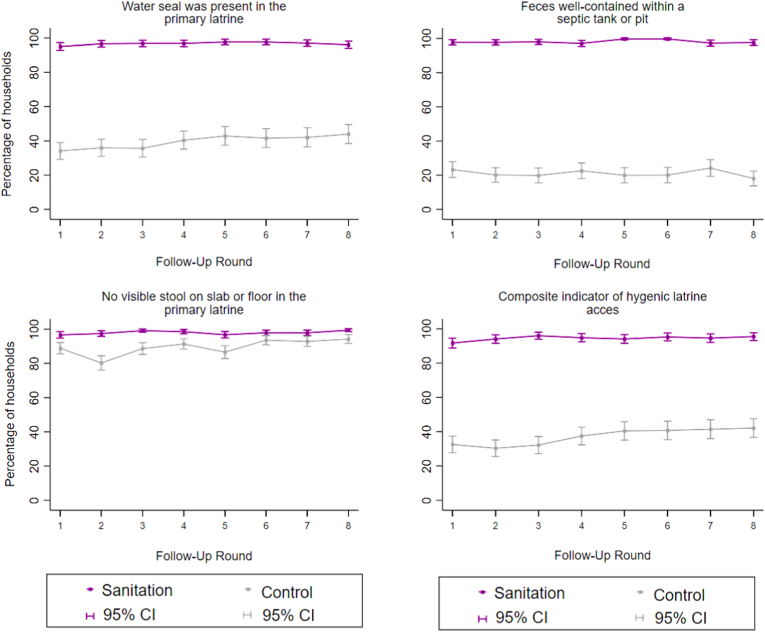
**Observed indicators of hygienic latrine access by study arm and round of data collection.** Composite indicator defined as the primary latrine observed to have a functional water seal, feces contained within a septic tank/pit and no visible feces on the slab or floor of the latrine. Data were collected between June 2014–December 2016, with each round completed over approximately three months. Intensity of intervention promotion was high during rounds 1–3 and low during rounds 4–6; there was no promotion during rounds 7–8. Each data collection round included approximately 720 households (360 per arm).

Observed indicators of hygienic latrine access were steady in the sanitation arm throughout the study period (Fig. 1), including periods with no ongoing promotion. The Cochran-Armitage test indicated no significant time trends in the sanitation arm (p = 0.10) while hygienic latrine access increased significantly among controls (p = 0.001). The difference between the sanitation and control arms in hygienic latrine access was somewhat smaller during the second half of the follow-up period (PD = 53.9, 95% CI: 47.9–60.0) than the first half (PD = 60.9, 95% CI: 55.2–66.8, p-value for interaction <0.001) and somewhat smaller during periods without promotion (PD = 53.2, 95% CI: 41.1, 62.7) than periods with promotion (PD = 59.8, 95% CI: 52.4–63.7, p-value for interaction = 0.21); however, this was driven by a modest improvement in hygienic latrine access in the control arm over time while access in the intervention arm remained steadily high around 94% throughout the study period (Table S1).

**Fig. 2:**
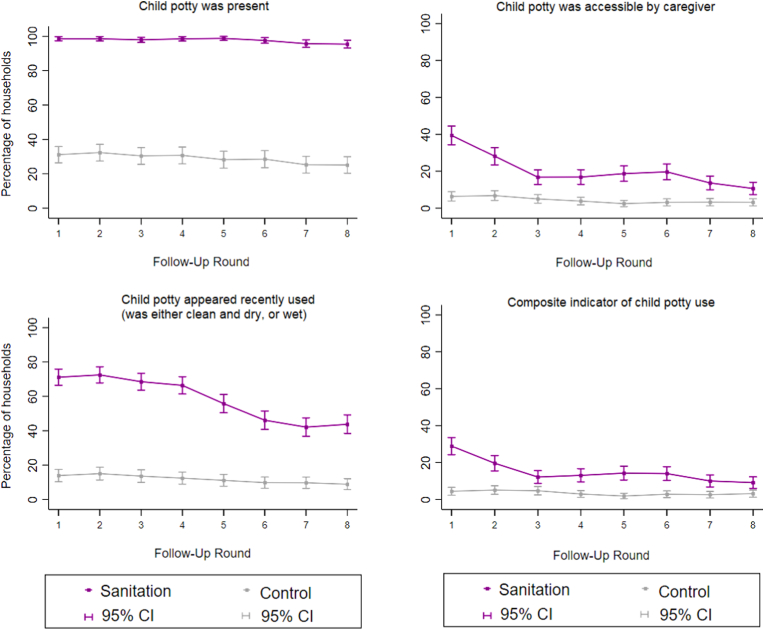
**Observed indicators of child potty use by study arm and round of data collection.** Composite indicator defined as a potty that was observed to be present, accessible by caregivers and appeared recently used. Data were collected between June 2014–December 2016, with each round completed over approximately three months. Intensity of intervention promotion was high during rounds 1–3 and low during rounds 4–6; there was no promotion during rounds 7–8. Each data collection round included approximately 720 households (360 per arm).

**Fig. 3:**
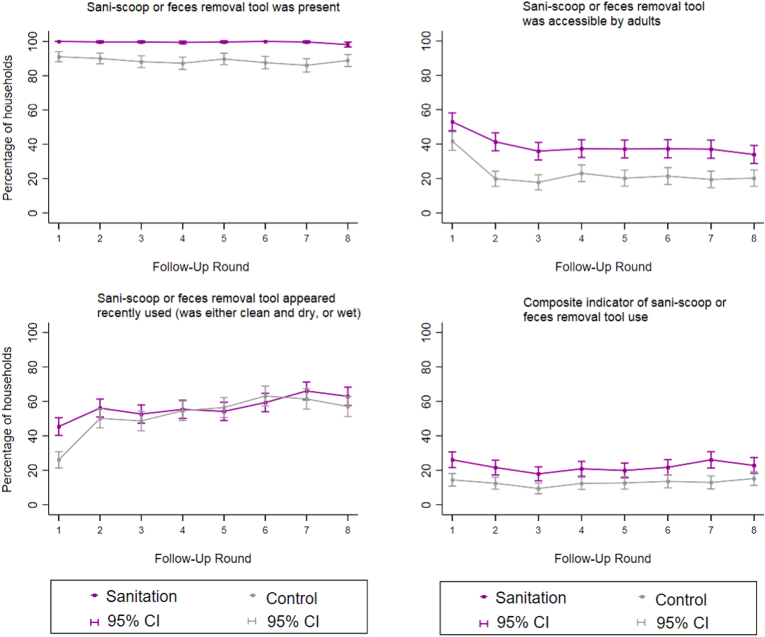
**Observed indicators of sani-scoop or feces removal tools use by study arm and round of data collection.** Composite indicator defined as a sani-scoop or other feces removal tool that was observed to be present, accessible by adults and appeared recently used. Data were collected between June 2014–December 2016, with each round completed over approximately three months. Intensity of intervention promotion was high during rounds 1–3 and low during rounds 4–6; there was no promotion during rounds 7–8. Each data collection round included approximately 720 households (360 per arm).

Observed indicators of child potty use declined in the sanitation arm after the first follow-up visit (Fig. 2). The Cochran-Armitage test indicated a significant decrease over time in both the sanitation arm (p = 0.0001) and control arm (p = 0.03). The difference between study arms in the prevalence of households with child potty use indicators was smaller during the second half of the follow-up period (PD = 9.3, 95% CI: 5.9–12.7) than the first half (PD = 14.3, 95% CI: 10.8–17.9, p-value for interaction = 0.002) and smaller during periods without promotion (PD = 9.0, 95% CI: 5.7, 12.3) than periods with promotion (PD = 14.5, 95% CI: 11.3–17.6, p-value for interaction <0.001) (Table S2). Indicators of sani-scoop/similar tool use remained steady over time (Fig. 3) with no evidence of time trends from the Cochran-Armitage test in either the sanitation arm (p = 0.80) and control arm (p = 0.43). The difference between study arms in the prevalence of households with indicators of sani-scoop/similar tool use was similar between the two halves of the study and between periods with and without promotion (Table S3).

### 3.4 Household factors associated with latrine, child potty and sani-scoop access and use

Compared to controls, the sanitation intervention increased hygienic latrine access more substantially among households where index children were below the median age (28 months), households where the caregiver or the father had no/only primary education vs. secondary/higher education, households with below-median vs. above-median wealth, and compounds with ≥10 residents and ≥2 children under 5 years (all interaction p-values <0.05, Table S1). These differences were driven by lower hygienic latrine access in the control arm among households with less education, less wealth and larger number of compound residents and children; hygienic latrine access was similar across these subgroups in the sanitation arm. For example, among households where the caregiver had no or primary education, hygienic latrine access increased by 73% (from 23% among controls to 96% in the sanitation arm) while among households where the caregiver had secondary or higher education, hygienic latrine access increased by 45% (from 48% to 93%). Similarly, hygienic latrine access increased by 75% (from 21% to 95%) among households with below-median wealth and by 41% (from 52% to 93%) among those with above-median wealth. Compared to controls, the intervention increased child potty use to a greater degree among households where children were <28 months old, and households located in compounds with ≥2 children under 5 years (interaction p-values <0.2, Table S2). There were no significant effect modifiers of intervention impacts on observed sani-scoop use (Table S3).

## 4 Discussion

In our substudy nested within a randomized controlled trial that provided sanitation hardware along with initial intensive behavior change promotion, the intervention led to a sustained increase in access to a hygienic latrine with a functioning water seal, feces well-contained in a septic tank or pit, and no feces on the slab or floor of the latrine 1–3.5 years after intervention initiation. Access remained high during periods with less intensive or no promotion. The gains in hygienic latrine access were more pronounced among households with less education, less wealth and larger number of residents and children. While the intervention significantly increased reported exclusive latrine use by adults and children 3–8 years old, reported exclusive latrine use by adults was high (77%) among controls, indicating existing sanitation norms and habits. The intervention increased availability of child feces management tools but their use remained low among intervention recipients, and any gains in potty use in the intervention arm declined over time.

The parent WASH Benefits study was an efficacy trial where latrine upgrades were provided to households free of charge and accompanied by initial intensive behavior change promotion. As such, the increase in hygienic latrine access among intervention recipients was substantially higher than what has typically been achieved by programmatic sanitation improvements. A systematic review on sustained adoption of sanitation interventions (defined as latrine ownership, presence, quality, functionality or use after the end of the intervention period) found that the most influential program factors associated with sustainability included frequent, personal contact with a health promoter and accountability over a period of time (K. Hulland et al., 2015). A study in Bangladesh, focused on areas that were previously declared “open defecation free” because all residents had installed latrines in response to a government program, found that 4.5 years later, households were more likely to have an improved or shared latrine if they received follow-up visits by a community health promoter about latrine use (Hanchett et al., 2011). Our study implemented intensive in-person promotion (6 visits/month) for the first two years after intervention initiation and less frequent promotion during the third year. No apparent decrease in hygienic latrine access after promotion activities tapered down or ceased suggests that functional hygienic latrines, once constructed, can be maintained without ongoing behavior change promotion. However, we note that our study was conducted in a setting with existing sanitation habits, as evidenced by high reported latrine use and secular increases in observed hygienic latrine access over time among controls. These existing norms may have supported ongoing maintenance of hygienic latrines among intervention recipients regardless of promotion activities.

Households in the sanitation arm also emptied the latrine pit less frequently, which may have been due to the recent construction of the pit latrine by the study, the dual-pit design which requires less frequent desludging, and slower pit fill-up due to reduced latrine sharing. Our findings are consistent with studies in Mozambique which found less frequent emptying of septic tanks at intervention compounds which received sanitation upgrades, including construction of sanitation infrastructure or subsidized provision of pour-flush toilets draining to a septic tank (Bauerl et al., 2016; Capone et al., 2020). In the latter of these two studies, the intervention was also significantly associated with increased hygienic emptying of septic tanks (Capone et al., 2020). In our study, unhygienic pit emptying practices (e.g., release into waterbodies) persisted among approximately a third of intervention recipients. We note that the delivered sanitation intervention did not include any hardware, facilities or behavioral messaging for safe emptying of pits. However, the double-pit design was intended to allow households to switch the super structure to the second pit when the first one filled up such that the contents of the first pit could decompose before being emptied. Many households in rural Bangladesh are located close to a pond, and intervention households may have continued to empty pits into water bodies out of convenience and habit. Future work should identify barriers against safe emptying of latrine pits to move up the sanitation ladder toward safely managed sanitation services.

Interventions substantially increased availability of child potties while the majority of households in both groups had a scoop or similar tool that can be used for removal of child feces. However, the use of these child feces management tools was low and inconsistent among intervention recipients. Similarly, in a recent trial in India that promoted safe child feces management along with broader latrine use, the intervention increased the percentage of households where the caregiver safely disposed of child feces by approximately 20 percentage points but only a third of intervention recipients practiced safe disposal (Caruso et al., 2022). Previous studies have identified latrine access and use by adults, and availability of child feces management tools as important determinants of safe child feces disposal (Ellis et al., 2020; Miller-Petrie et al., 2016). In our study, child feces management products as well as latrines were provided for free, and latrine use among adults was high among intervention recipients, suggesting additional barriers to child feces management. Such barriers may include lack of perceived risk from child feces, time needed for child feces management and competing demands on caregivers’ time for household tasks or income-generating activities, and social norms (Ellis et al., 2020; K. R. Hulland et al., 2015; Mbuya and Humphrey, 2016; Miller-Petrie et al., 2016).

In rural Bangladesh, potties are uncommon and not widely available for purchase (Sultana et al., 2013), and rural parents are typically not aware about the benefits of using potties or may not know how to train their children to use the potty (Hussain et al., 2017). Stool of young children is typically considered harmless or less harmful and less disgusting than adult feces in South Asia (Brown et al., 2013), and therefore, consistently using a child potty for child defecation and scoop for child feces disposal may be less prioritized. The potties and scoops provided as part of the WASH Benefits interventions were iteratively piloted and tailored with community input, using the integrated behavioral model for water, sanitation and hygiene (IBM-WASH) (Dreibelbis et al., 2013). During the piloting stage, semi-structured interviews, group discussions, and observations among 26 households in the study area indicated that caregivers found the potties acceptable and reported time savings from using a potty for child feces management (Hussain et al., 2017). Some children refused to defecate in the potty, and younger children (<1 year old) were too small to sit on it and had to be held (Hussain et al., 2017). The behavior change program provided along with the WASH Benefit interventions included discussions and activities on potty familiarization, potty training, problems children encountered while defecating in the potty, potty cleaning and maintenance, benefits and barriers of using potty, and feces disposal location (Hussain et al., 2017). Future studies should investigate additional strategies to promote safe child feces management practices in low-income settings.

Our previous work in rural Bangladesh suggested that unsafe child feces disposal is associated with increased *E. coli* contamination of child hands and stored drinking water but showed no clear associations with child gastrointestinal illness (Islam et al., 2018, 2020). A study in rural India found higher levels of *E. coli* on the floor or ground after a child defecated on these surfaces and the feces were removed, and on tools used to dispose of the feces even after cleaning (Bauza et al., 2020). In the same study, unsafe child feces disposal was associated with increased levels of *E. coli* in stored drinking water and on caregiver hands (Bauza et al., 2020). A microbial source tracking study in urban slums in Kenya found that young children's feces were the main source of fecal contamination detected inside the home environment, while contamination detected outside the home was more commonly from feces of adults and older children (Bauza et al., 2019). Out of five studies reviewed in a recent meta-analysis, two found that safe child defecation and safe feces disposal was associated with a reduction in diarrhea while the others did not find an association (Morita et al., 2016). In a study in rural Bangladesh, children in households where caregivers reported unsafe child feces disposal had higher environmental enteropathy scores and odds of being wasted (George et al., 2016). A study in Mozambique suggested that deposition of a small amount of child feces onto soil can support ongoing transmission of *Ascaris* infections (Capone et al., 2022). These findings suggest that child feces are a potential source of fecal pathogens in the environment that could contribute to adverse effects on child health. Sanitation programs that solely focus on the feces of adults and older children are unlikely to reduce fecal contamination in the home environment without measures for hygienic defecation and feces disposal for young children. Interventions aiming to reduce fecal exposure should continue to develop and test approaches to reduce child feces in the environment, alone or combined with broader sanitation improvements (Caruso et al., 2022; Sclar et al., 2022).

Our study had some limitations. While the observable uptake indicators objectively reflected the availability and condition of infrastructure and supplies they may not accurately represent actual use as availability does not ensure use. However, our observed indicators included visual signs of likely recent use, such as the tools being wet or free of dust. Additionally, our study had a longitudinal design where we visited the same households several times. Therefore, it is possible that anticipation of a data collection visit might alert participants to improve their practices during the data collection period and, therefore, overestimate uptake (Arnold et al., 2015). We attempted to reduce this limitation by arriving unannounced to minimize reactivity (Cousens et al., 1996). Another limitation is that the intervention was delivered under optimal conditions during an efficacy trial, and so these findings do not readily generalize to routine programs with more limited resources.

## 5 Conclusion

In a randomized controlled trial that provided latrine upgrades along with initial intensive behavior change promotion, high access to hygienic latrines with a functional water seal and well-contained fecal waste was sustained among intervention recipients up to 3.5 years after intervention initiation. Access was high even after behavior change promotion stopped. Our findings from a setting with existing sanitation norms indicate that latrine quality, once established, can be maintained without ongoing promotion activities. Future studies should investigate strategies to achieve and sustain access to and use of latrines that effectively isolate feces from the environment in settings with different baseline sanitation norms. Despite free provision of potties and sani-scoop tools and messages on safe child feces management included in the behavior change promotion program, adoption of these tools remained low among intervention recipients. Studies should investigate barriers to safe child feces management practices and strategies to ensure their sustained adoption, as well as test the effectiveness of child feces management interventions, alone or combined with broader sanitation improvements, in reducing fecal exposures.

## Funding sources

Bill & Melinda Gates Foundation, National Institutes of Health (NIH).

## Data statement

De-identified data used for this analysis will be made freely available on OSF upon publication (https://osf.io/6u7cn/).

## Declaration of competing interest

We declare no conflicts of interest.
