# Supplementary material for: Assessing sustained uptake of latrine and child feces management interventions: Extended follow-up of a cluster-randomized controlled trial in rural Bangladesh 1–3.5 years after intervention initiation
Source: Int J Hyg Environ Health. 2023 May;250:114149. doi: 10.1016/j.ijheh.2023.114149 (PMC10186382; doi:10.1016/j.ijheh.2023.114149)
Supplement: Multimedia component 1 [file mmc1.docx]

Supporting Information

Table S1: Effect modifiers of intervention impact on hygienic latrine access ^a^

|  | Sanitation | | Control | | Sanitation vs. Control | Interaction  p-value ^b^ |
| --- | --- | --- | --- | --- | --- | --- |
|  | N | % (n) | N | % (n) | PD (95% CI) |  |
| Time since study onset | | | | | | |
| First half (rounds 1-4) | 1403 | 93.6 (1314) | 1391 | 33.0 (460) | 60.9 (55.2, 66.8) | **<0.001** |
| Second half (rounds 5-8) | 1332 | 94.3 (1256) | 1267 | 41.1 (520) | 53.9 (47.9, 60.0) |  |
| Behavior change promotion | | | | | | |
| Ongoing (rounds 1-6) | 2076 | 94.3 (1957) | 2032 | 34.5 (702) | 59.8 (52.4, 63.7) | 0.21 |
| None (rounds 7-8) | 659 | 95.0 (626) | 625 | 41.8 (261) | 53.2 (41.1, 62.7) |  |
| Index child age at follow-up | | | | | | |
| <28 months | 1339 | 94.3 (1262) | 1361 | 32.3 (439) | 62.5 (56.6, 68.3) | **<0.001** |
| ≥28 months | 1396 | 93.7 (1308) | 1296 | 41.7 (541) | 52.6 (46.2, 58.9) |  |
| Caregiver age | | | | | | |
| < 23 yrs | 1273 | 92.5 (1178) | 1241 | 38.7 (460) | 53.8 (47.0, 60.6) | 0.27 |
| ≥ 23 yrs | 1462 | 95.2 (1392) | 1416 | 35.0 (520) | 59.6 (53.0, 66.2) |  |
| Education of caregiver | | | | | | |
| Secondary or above | 1525 | 93.3 (1423) | 1474 | 47.8 (704) | 45.2 (37.8, 52.5) | **<0.001** |
| None or primary | 1210 | 95.9 (1154) | 1184 | 23.4 (276) | 72.9 (67.0, 79.2) |  |
| Education of father | | | | | | |
| Secondary or above | 1130 | 92.3 (1043) | 1129 | 46.6 (526) | 45.0 (36.3, 53.3) | **<0.001** |
| None or primary | 1605 | 95.1 (1527) | 1529 | 29.7 (454) | 65.9 (60.8, 72.9) |  |
| Household wealth | | | | | | |
| Above median | 1356 | 92.7 (1257) | 1386 | 51.6 (715) | 41.0 (33.1, 48.7) | **<0.001** |
| Below median | 1379 | 95.2 (1313) | 1272 | 20.8 (265) | 75.4 (69.5, 81.4) |  |
| Number of people in compound | | | | | | |
| ≥10 people | 1596 | 94.2 (1503) | 1400 | 31.9 (446) | 62.3 (57.1, 67.5) | **0.004** |
| <10 people | 1139 | 93.7 (1067) | 1258 | 42.5 (534) | 50.0 (42.0, 57.9) |  |
| Number of children <5 yrs in compound | | | | | | |
| ≥2 children | 1499 | 93.0 (1394) | 1540 | 32.9 (507) | 62.2 (55.2, 69.2) | **0.02** |
| <2 children | 1236 | 95.1 (1176) | 1118 | 42.3 (473) | 51.5 (44.4, 58.7) |  |

PD: Prevalence difference; CI: Confidence interval

^a^ **Composite indicator of hygienic latrine access** defined as the primary latrine observed to have a functional water seal, feces contained within a septic tank/pit and no visible feces on the slab or floor of the latrine.

^b^ Values in bold indicate interaction terms statistically significant at the p<0.2 level.

**Table S2: Effect modifiers of intervention impact on potty use ^a^**

|  | Sanitation | | Control | | Sanitation vs. Control | Interaction  p-value ^b^ |
| --- | --- | --- | --- | --- | --- | --- |
|  | N | % (n) | N | % (n) | PD (95% CI) |  |
| Time since study onset | | | | | | |
| First half (rounds 1-4) | 1403 | 18.5 (260) | 1391 | 4.3 (60) | 14.3 (10.8, 17.9) | **0.002** |
| Second half (rounds 5-8) | 1332 | 11.8 (158) | 1266 | 2.6 (33) | 9.3 (5.9, 12.7) |  |
| Behavior change promotion | | | | | | |
| Ongoing (rounds 1-6) | 2076 | 17.1 (355) | 2032 | 3.7 (75) | 14.5 (11.3, 17.6) | **<0.001** |
| None (rounds 7-8) | 659 | 9.6 (63) | 625 | 2.9 (18) | 9.0 (5.7, 12.3) |  |
| Index child age at follow-up | | | | | | |
| <28 months | 1339 | 19.1 (256) | 1361 | 4.3 (58) | 15.0 (11.2, 18.7) | **0.001** |
| ≥28 months | 1396 | 11.6 (162) | 1296 | 2.7 (35) | 8.9 (5.7, 12.1) |  |
| Caregiver age | | | | | | |
| < 23 yrs | 1273 | 15.0 (192) | 1241 | 4.2 (52) | 11.5 (7.9, 15.1) | 0.69 |
| ≥ 23 yrs | 1462 | 15.6 (226) | 1416 | 2.9 (41) | 12.3 (8.6, 16.0) |  |
| Education of caregiver | | | | | | |
| Secondary or above | 1525 | 16.2 (248) | 1474 | 5.4 (80) | 10.7 (6.6, 14.7) | 0.24 |
| None or primary | 1210 | 14.1 (170) | 1183 | 1.1 (13) | 13.3 (9.9, 16.7) |  |
| Education of father | | | | | | |
| Secondary or above | 1130 | 16.6 (188) | 1129 | 6.1 (69) | 11.3 (7.2, 15.5) | 0.64 |
| None or primary | 1605 | 14.3 (230) | 1528 | 1.5 (24) | 12.3 (9.0, 15.6) |  |
| Household wealth | | | | | | |
| Above median | 1356 | 16.2 (220) | 1385 | 4.7 (66) | 12.0 (7.8, 16.2) | 0.90 |
| Below median | 1379 | 14.3 (198) | 1272 | 2.1 (27) | 11.7 (8.3, 15.1) |  |
| Number of people in compound | | | | | | |
| ≥10 people | 1596 | 15.9 (254) | 1400 | 2.7 (38) | 12.8 (9.3, 16.2) | 0.39 |
| <10 people | 1139 | 14.4 (164) | 1257 | 4.4 (55) | 10.7 (6.4, 15.0) |  |
| Number of children <5 yrs in compound | | | | | | |
| ≥2 children | 1499 | 16.9 (254) | 1539 | 2.3 (36) | 14.5 (10.9, 18.2) | **0.01** |
| <2 children | 1236 | 13.3 (164) | 1118 | 5.1 (57) | 8.9 (5.7, 12.2) |  |

PD: Prevalence difference; CI: Confidence interval

^a^ **Composite indicator of potty use** defined as a potty that was observed to be present, accessible by caregivers and appeared recently used.

^b^ Values in bold indicate interaction terms statistically significant at the p<0.2 level.

**Table S3: Effect modifiers of intervention impact on sani-scoop use ^a^**

|  | Sanitation | | Control | | Sanitation vs. Control | Interaction  p-value ^b^ |
| --- | --- | --- | --- | --- | --- | --- |
|  | N | % (n) | N | % (n) | PD (95% CI) |  |
| Time since study onset | | | | | | |
| First half (rounds 1-4) | 1403 | 21.6 (304) | 1391 | 12.2 (170) | 9.5 (1.8, 17.2) | 0.92 |
| Second half (rounds 5-8) | 1332 | 22.6 (301) | 1266 | 13.5 (172) | 9.3 (1.9, 16.8) |  |
| Behavior change promotion | | | | | | |
| Ongoing (rounds 1-6) | 2076 | 21.4 (444) | 2032 | 12.5 (254) | 9.4 (3.0, 15.8) | **0.89** |
| None (rounds 7-8) | 659 | 24.4 (161) | 625 | 14.1 (88) | 9.1 (2.7, 15.5) |  |
| Index child age at follow-up | | | | | | |
| <28 months | 1339 | 22.2 (295) | 1361 | 12.6 (171) | 9.3 (1.6, 17.0) | 0.93 |
| ≥28 months | 1396 | 22.4 (310) | 1296 | 13.1 (171) | 9.6(1.8, 17.3) |  |
| Caregiver age | | | | | | |
| < 23 yrs | 1273 | 23.6 (297) | 1241 | 13.0 (163) | 10.9 (2.8, 19.0) | 0.44 |
| ≥ 23 yrs | 1462 | 21.0 (308) | 1416 | 13.0 (179) | 8.1 (0.1, 16.2) |  |
| Education of caregiver | | | | | | |
| Secondary or above | 1525 | 20.8 (317) | 1474 | 12.5 (184) | 8.6 (1.5, 17.9) | 0.92 |
| None or primary | 1210 | 23.8 (288) | 1183 | 13.4 (158) | 9.2 (0.1, 18.3) |  |
| Education of father | | | | | | |
| Secondary or above | 1130 | 19.5 (220) | 1129 | 14.2 (160) | 6.3 (-2.2, 15.8) | 0.33 |
| None or primary | 1605 | 24.0 (385) | 1528 | 11.9 (182) | 11.3 (3.1, 19.6) |  |
| Household wealth | | | | | | |
| Above median | 1356 | 20.0 (271) | 1385 | 14.2 (197) | 7.9 (-0.4, 16.2) | 0.47 |
| Below median | 1379 | 24.2 (334) | 1272 | 11.4 (145) | 11.0 (2.5, 19.4) |  |
| Number of people in compound | | | | | | |
| ≥10 people | 1596 | 20.1 (321) | 1400 | 13.1 (183) | 7.4 (-0.3, 15.1) | 0.30 |
| <10 people | 1139 | 24.9 (284) | 1257 | 12.6 (159) | 12.0 (2.6, 21.4) |  |
| Number of children <5 yrs in compound | | | | | | |
| ≥2 children | 1499 | 21.4 (320) | 1539 | 11.0 (169) | 10.4 (3.1, 17.7) | 0.55 |
| <2 children | 1236 | 23.1 (285) | 1118 | 15.5 (173) | 8.1 (-1.1, 17.2) |  |

PD: Prevalence difference; CI: Confidence interval

^a^ **Composite indicator of sani-scoop use** defined as a sani-scoop or other feces removal tool that was observed to be present, accessible by adults and appeared recently used.

^b^ Values in bold indicate interaction terms statistically significant at the p<0.2 level.

**
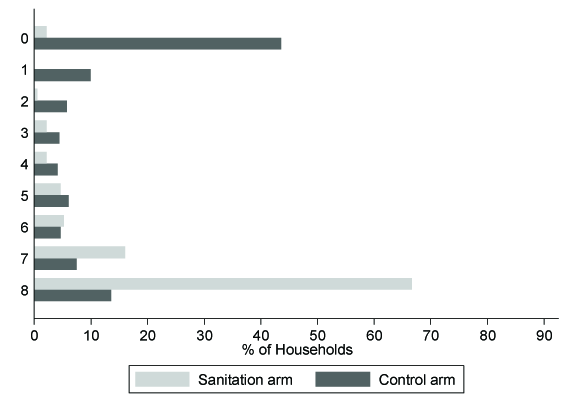
**

**Figure S1: Frequency distribution for the number of data collection rounds (out of eight) households had observed indicators of hygienic latrine access**

**
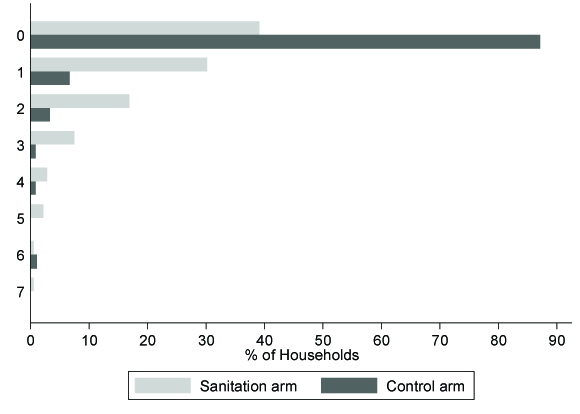
**

**Figure S2: Frequency distribution for the number of data collection rounds (out of eight) households had observed indicators of potty use**


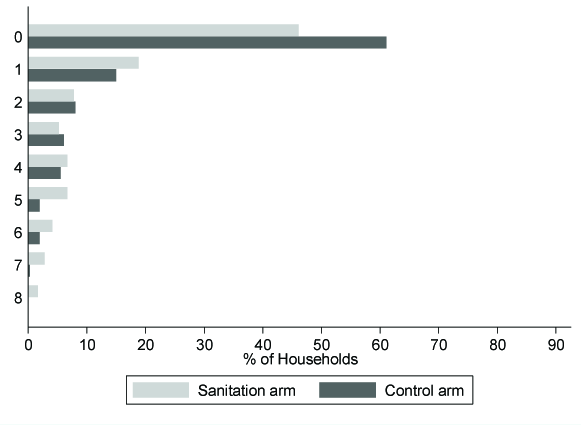


**Figure S3: Frequency distribution for the number of data collection rounds (out of eight) households had observed indicators of sani-scoop use**
